# The Role of *RYR2* in Atrial Fibrillation

**DOI:** 10.1155/2023/6555998

**Published:** 2023-03-15

**Authors:** Bernhard M. Boehm, Jochen Gaa, Petra Hoppmann, Eimo Martens, Dominik S. Westphal

**Affiliations:** ^1^Institute of Human Genetics, School of Medicine & Klinikum rechts der Isar, Technical University of Munich, Germany; ^2^Department of Diagnostic and Interventional Radiology, School of Medicine & Klinikum rechts der Isar, Technical University Munich, Germany; ^3^Department of Internal Medicine I, School of Medicine & Klinikum rechts der Isar, Technical University of Munich, Germany

## Abstract

*Background*. Atrial fibrillation (AF) is a common arrhythmia in elderly patients and is associated with increased risk of mortality. The pathogenesis of AF is complex and based on multiple genetic and environmental factors. Genome-wide association studies identified several loci in AF patients, indicating the complex genetic architecture of this disease. In rare cases, familial forms of AF have been described. Today, pathogenic variants in at least 11 different genes are associated with monogenic AF. *Case presentation*. The 37-year-old male patient presented to our emergency department with AF. At the age of 35, he had already been diagnosed with paroxysmal AF. Additionally, his 34-year-old brother had also been diagnosed with AF as well as nonobstructive hypertrophic cardiomyopathy. Moreover, the patient's father was diagnosed with AF in his twenties. Transthoracic echocardiography and cardiac MRI revealed a reduced systolic left ventricular ejection without any signs of hypertrophic cardiomyopathy. Genetic testing identified the heterozygous missense variants c.3371C > T, p.(Pro1124Leu) in *RYR2* (NM_001035.3) and c.2524C > A, p.(Pro842Thr) in *HCN4* (NM_005477.3) in the patient's and his brother's DNA. *Discussion*. This case of familial AF helps to strengthen the role of *RYR2* as a disease gene in the context of AF. Although the variant in *RYR2* needs to be classified formally as variant of unknown significance, we regard it as probably disease-causing due to the previously published data. As *RYR2* has already been identified as a possible target for prevention and therapy of AF, the knowledge of variants in *RYR2* might become even more crucial for individual molecular therapies in the future.

## 1. Introduction

Atrial fibrillation (AF) is a common arrhythmic disease, affecting an estimate of approximately 33 million patients globally [1]. It is characterized by irregular and often increased heart rate. Arterial hypertension, diabetes mellitus, advancing age, adiposity, and cardiovascular disease are reported amongst predisposing factors for developing AF. Complications of AF include thromboembolic stroke, heart failure, dementia, and increased risk of death [2, 3].

The pathogenesis of AF is influenced by genetic and environmental factors. A previous study has shown familial clustering of patients with lone AF [4], while a study on monozygotic twins suggested a heritability of AF as high as about 60% [5]. Therefore, a strong genetic component could be early assumed. This assumption was confirmed by modern genome-wide association studies (GWAS) which are based on the analysis of single-nucleotide polymorphisms in large patient and control cohorts. One of these studies estimated the heritability of AF in patients of European descent at about 22% [6]. Additionally, following GWAS identified almost 140 different AF loci in participants of predominantly European ancestry [7, 8], emphasizing the complex underlying genetic architecture of this common disease.

While most cases of AF are based on interaction of multiple genetic and environmental factors, familial forms of AF have also been described in rare cases. Today, the Online Mendelian Inheritance in Man (OMIM) database lists 11 different genes in which pathogenic variants have a distinct association with familial AF (https://www.omim.org/, last accessed: 02/23). Most of these genes are coding for ion channels and their associated forms of AF are inherited in an autosomal-dominant manner. Some of these genes are associated with other inborn arrhythmias, like the well-known long QT syndrome (LQTS). The first described variant of familial AF, for example, was caused by variants in *KCNQ1*. The loss-of-function of KCNQ1 leads to LQTS type 1, while the gain function causes shortening of the atrial refractory period, which in turn increases the risk of developing AF [9]. In this case study, we report two brothers, in which a missense variant in *RYR2* might explain early-onset AF combined with hypertrophic cardiomyopathy (HCM) in one of the siblings.

## 2. Case Presentation

**Figure 1:**
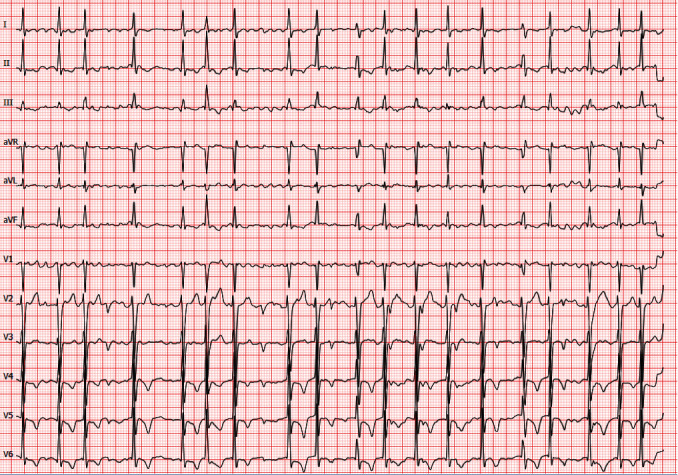
Resting ECG at admission. Tachycardic atrial fibrillation with irregular rhythm at a heart rate of 111 beats per minute. Repolarization showed signs of left ventricular hypertrophy.

**Figure 2:**
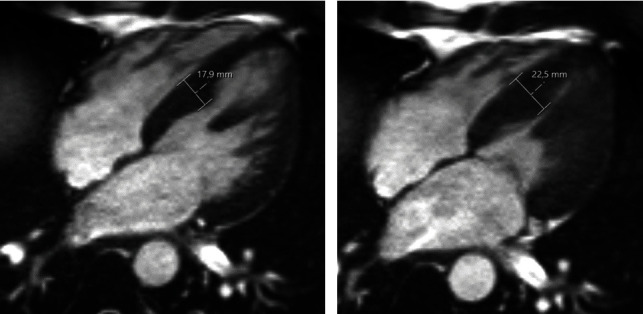
Cardiac MRI of the patient's brother (1.5 Tesla, cine sequence). (a) End-diastolic septal myocardial hypertrophy with maximum measured septum thickness of 17.9 mm. (b) End systolic septal myocardial hypertrophy with maximum measured septum thickness of 22.5 mm.

**Figure 3:**
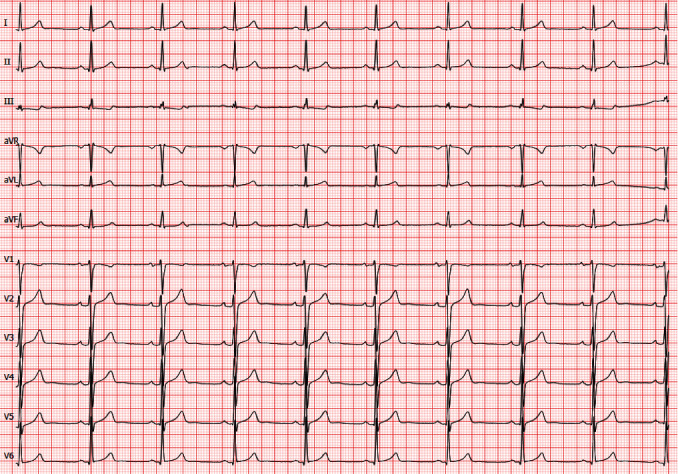
Resting ECG at 1.5-year follow-up. Regular sinus rhythm at a heart rate of 55 beats per minute with persistent signs of left ventricular hypertrophy.

A 37-year-old male patient presented to our emergency department with dyspnea, palpitations, and AF (Figure 1). Paroxysmal AF had already been diagnosed 2 years prior. The patient's family history was positive for AF. The 34-year-old brother had also been diagnosed with persistent AF as well as HCM (Figure 2). His father was affected by AF with onset in his twenties. There were no further affected family members reported. Transthoracic echocardiography (TTE) of the patient revealed a reduced systolic left ventricular ejection fraction (LVEF) of 40%. Medication for heart rate control and heart failure consisting of bisoprolol, ramipril, spironolactone, and torasemide was started while already taken oral anticoagulation with apixaban was continued. Coronary artery disease was excluded via cardiac catheter examination. Additionally, performed cardiac MRI confirmed the reduced LVEF, however, without any signs of HCM (end-diastolic interventricular septum thickness: 11 mm) despite repolarization abnormalities in the initial resting electrocardiogram (ECG, Figure 1). Eventually, pulmonary vein isolation (PVI) was done, but there was an early relapse after a few days. Since treatment with amiodarone failed to restore sinus rhythm, electrical cardioversion was performed. Asymptomatic AF recurred again, shortly after the executed intervention, and the patient was discharged from our clinic with a long-term amiodarone therapy. A week later, the patient presented to our hospital again because of increasing palpitations and fatigue. The heart rhythm was converted to sinus rhythm by external electrical cardioversion. At 6-month follow-up, the patient reported a reduction of symptoms under amiodarone and heart failure medication. Long-term ECG showed sinus rhythm without any signs of AF. TTE revealed an increase of LVEF to 50%. Diuretic medication (spironolactone, torasemide) and amiodarone were discontinued. At 1.5-year follow-up, the patient was asymptomatic without any signs of AF in resting (Figure 3) and long-term ECG. Oral anticoagulation as well as heart failure medication consisting of bisoprolol and ramipril have been continued until today.

Additionally performed DNA analysis by duo exome sequencing identified the heterozygous missense variants c.3371C > T, p.(Pro1124Leu) in *RYR2* (NM_001035.3) and c.2524C > A, p.(Pro842Thr) in *HCN4* (NM_005477.3) in the patient's and his brother's DNA. Testing of the parents for the variants was recommended but declined until today. There are no further siblings for segregation analysis. Since catecholaminergic polymorphic ventricular tachycardia (CPVT) was reported in patients with pathogenic variants in *RYR2*, an exercise ECG examination was done in the patient and his brother but did not reveal any signs of exercise-triggered arrhythmia.

## 3. Discussion

*RYR2* encodes the cardiac ryanodine receptor 2 (RYR2), an ion channel protein located in the membrane of the sarcoplasmic reticulum (SR). The receptor is fundamental for cardiac muscle contraction by releasing calcium ions from SR to the cytosol and is activated by inward flow of calcium ions via L-type calcium channels in close proximity [10]. Pathogenic variants in *RYR2* variants have been associated with different cardiac diseases in the OMIM database, namely the arrhythmogenic right ventricular cardiomyopathy type 2 (ARVC2, OMIM #600996), the catecholaminergic polymorphic ventricular tachycardia type 1 (CPVT1, OMIM #604772), and ventricular arrhythmias due to cardiac ryanodine receptor calcium release deficiency syndrome (VACRDS, OMIM #115000) (last accessed: 02/23). Moreover, variants in *RYR2* have been identified in patients with HCM [11] as well as AF [12, 13].

The hereby reported heterozygous missense variant p.(Pro1124Leu) in *RYR2* has also already been identified in a patient with HCM. Following functional cell studies of the variant showed that it leads to a cytosolic loss-of-function and a luminal gain-of-function of the RYR2. Knock-in mice carrying the variant demonstrated a mild HCM phenotype with homozygous carriers being more affected than the heterozygous ones. Additionally, an increased susceptibility for cardiac arrhythmia could be detected in these mice. The authors suggested that the observed phenotype was caused by increased expression of calmodulin [14]. The z-score of *RYR2* in the Genome Aggregated Database (gnomAD) is 5.78 indicating a high intolerance for missense variants, although it is not an ultra-rare variant since the variant's minor allele frequency is 0.003% (7 heterozygous carriers, https://gnomad.broadinstitute.org/, last accessed: 02/23). However, the *in silico* prediction score “Combined Annotation Dependent Depletion” (CADD) for the reported variant is 32 emphasizing its possible disease-causing effect. Furthermore, three other missense variants in *RYR2* that were associated with CPVT in humans (p.(Arg2474Ser), p.(Arg2386Ile), p.(Leu433Pro)) have been associated with increased susceptibility for AF in mouse models [15].

Exome sequencing also identified the rare heterozygous missense variant c.2524C > A, p.(Pro824Thr) in *HCN4* (NM_005477.3) in both brothers. *HCN4* encodes a hyperpolarization-activated cyclic nucleotide-gated ion channel that is expressed in the heart and the brain [16]. *HCN4* variants have been associated with sick sinus syndrome type 2 (OMIM #163800) in which AF could be observed [17]. *HCN4* has also been identified as a susceptibility locus for AF in a GWAS [18]. Nevertheless, the *HCN4* variant found in our patient was not reported before. The variant was therefore classified as variant of unknown significance (VUS).

We cannot exclude a contribution of the variant in *HCN4* to the AF, and the variant in *RYR2* needs to be classified formally as VUS. Nevertheless, we regard the missense variant c.3371C > T, p.(Pro1124Leu) in *RYR2* (NM_001035.3) as most likely disease-causing due to the previously published data. This reported case helps to strengthen the role of *RYR2* as a disease gene in context of AF. The knowledge of *RYR2* variants in patients with AF might become even more crucial for individual molecular therapies, as the RYR2 has already been identified as possible target of prevention and treatment of AF [19, 20].

## Data Availability

The data that support the findings of this study are available from the corresponding author upon reasonable request.
